# Does Bone Density Affect Outcomes in Lateral Lumbar Interbody Fusion? A Propensity Score-Matched Analysis of Preoperative Hounsfield Units

**DOI:** 10.3390/jcm13216374

**Published:** 2024-10-24

**Authors:** Akihiko Hiyama, Daisuke Sakai, Hiroyuki Katoh, Masato Sato, Masahiko Watanabe

**Affiliations:** Department Orthopaedic Surgery, Tokai University School of Medicine, 143 Shimokasuya, Isehara 259-1193, Kanagawa, Japan; daisakai@is.icc.u-tokai.ac.jp (D.S.); hero@tokai.ac.jp (H.K.); sato-m@is.icc.u-tokai.ac.jp (M.S.); masahiko@is.icc.u-tokai.ac.jp (M.W.)

**Keywords:** low back pain, lateral lumbar interbody fusion, indirect decompression, patient-reported outcome measures, Hounsfield units, propensity score matching

## Abstract

**Background**: This study aimed to assess whether preoperative Hounsfield unit (HU) values differ in short-term clinical outcomes after lateral lumbar interbody fusion (LLIF) surgery. **Methods**: In a retrospective analysis, 109 patients undergoing LLIF for lumbar degenerative diseases (LDD) were reviewed. Preoperative Computed Tomography (CT) scans measured HU values at the L1–L4 vertebrae, dividing patients into low and high HU groups. After conducting a cluster analysis of preoperative Hounsfield unit (HU) values, patients were categorized into low and high HU groups using propensity score matching (PSM). The outcomes measured one-year post-surgery included pain intensity (Numeric Rating Scales for Low Back Pain (NRS_LBP_), Leg Pain (NRS_LP_), and Leg Numbness (NRS_LN_)) and quality of life (Japanese Orthopedic Association Back Pain Evaluation Questionnaire: JOABPEQ). **Results**: After PSM, there were 26 patients in each group. Significant improvements were noted in both low and high HU groups post-surgery, with the low HU group showing a decrease in NRS_LBP_ from 6.2 to 3.7, NRS_LP_ from 7.4 to 2.5, and NRS_LN_ from 6.4 to 3.0. The high HU group exhibited similar improvements (NRS_LBP_: 6.5 to 3.6, NRS_LP_: 6.3 to 2.5, NRS_LN_: 6.2 to 2.4). JOABPEQ scores improved significantly in both groups across all domains, with no significant differences observed. Preoperative HU values have little correlation with the short-term outcomes of pain and quality of life in LLIF surgery. **Conclusions**: This study suggests reconsidering the role of HU values following indirect decompression via LLIF, particularly in evaluating pain and patient-reported outcome measures in patients with LDD.

## 1. Introduction

Addressing osteoporosis in spinal surgery patients is crucial for preventing postoperative complications. It is reported that osteoporosis affects 20% of spinal surgery patients, a figure that increases to 50% in women over 50 years of age [1,2]. A recommendation by the International Society for Clinical Densitometry suggested that surgeons should evaluate bone health in males aged ≧ 70 years and females aged ≧ 65 years who undergo spine surgery [3]. However, intriguingly, only approximately 20% of spinal surgeons routinely screen for osteoporosis before surgery [1]. Another study highlighted that while 60% of surgeons acknowledge the need to modify surgical methods in the presence of osteoporosis, only 4% actually conduct quantitative evaluations of bone mineral density (BMD) preoperatively [4]. The background of these research results is that there is a large discrepancy between osteoporosis management and actual implementation [5]. Although dual-energy X-ray absorptiometry (DXA) is considered the gold standard for evaluating osteoporosis [6,7], it has limitations like inconsistent readings at different sites and an inability to assess cortical bone.

In this context, CT scan-based methods for analyzing bone density have emerged as a viable alternative [6,8]. Some papers have already compared Hounsfield units (HU) and the DXA of spine CT scans and reported the correlation between HU and BMD values [9,10,11]. For spinal surgeons, HU measurement from CT images is advantageous during routine preoperative planning for lumbar surgery, eliminating the need for additional testing.

Rapid, user-friendly, and readily available, HU evaluation is gaining support through systematic review studies [11]. HU assessment can be particularly beneficial in lumbar deformities, where DXA-measured BMD might be inaccurately elevated due to bone spurs [8].

In the realm of surgical techniques, the role of indirect decompression via LLIF is well recognized [12,13,14]. LLIF is increasingly acknowledged as a minimally invasive spinal surgery option, as it allows for indirect decompression without damaging posterior supporting elements, offering a less invasive alternative to traditional anterior spinal fusion. However, the failure of indirect decompression is also known. Factors such as osteoporosis, cage subsidence, and intraoperative endplate injury could influence the success of this approach [15,16]. Bone health in spine surgery has attracted wide attention since it may influence the outcome of the surgery [17]. In patients with osteoporosis, the causes of poor short-term outcomes after LLIF surgery may include insufficient immediate stability of the implant, increased risk of postoperative complications, delayed early functional recovery, and difficulty in pain management. Therefore, for patients with osteoporosis, it is necessary to consider these risks in preoperative evaluation and planning and to implement appropriate measures (e.g., bone strengthening therapy, careful postoperative management, and appropriate pain management). Previous research has shown that low HU values significantly increase the risk of future osteoporotic vertebral fractures [18,19]. Regarding surgical complications, low HU values have been linked to postoperative complications such as adjacent segment fractures and cage subsidence [20,21,22,23]. However, the impact of low HU values on short-term clinical outcomes assessed using patient-reported outcome measures following LLIF surgery has yet to be widely reported. The aim of this study is to investigate whether preoperative HU values, measured via CT, influence short-term clinical outcomes such as postoperative pain and quality of life following LLIF surgery in patients with LDD. By understanding this relationship, we seek to provide insights into the potential role of HU values in preoperative planning and postoperative management for LLIF patients.

## 2. Materials and Methods

### 2.1. Ethics Approval

We had the study protocol reviewed and approved by the Committee on Ethics, the Institutional Review Board, and the Profit Reciprocity Committee (23R-189). Because this study was retrospective, the requirement for informed consent was waived.

### 2.2. Included Patients

**Figure 1:**
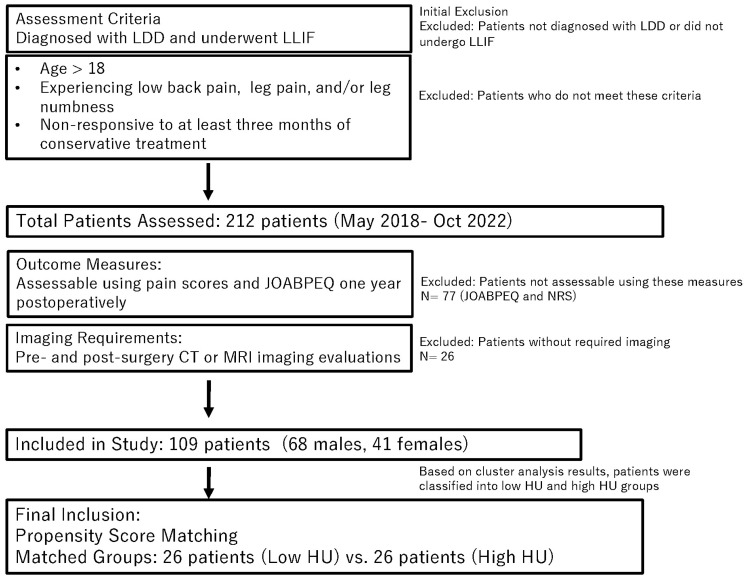
Flow chart of patient assessment, exclusion, and inclusion. LDD; lumbar degenerative disease, LLIF; lateral lumbar interbody fusion, NRS; Numeric Rating Scale, JOABPEQ; Japanese Orthopedic Association Back Pain Evaluation Questionnaire, HU; Hounsfield unit, CT; Computed tomography, MRI; magnetic resonance imaging.

The patient selection process is illustrated in Figure 1 (flow chart).

A retrospective review was conducted for patients diagnosed with lumbar degenerative diseases (LDD) who underwent LLIF with posterior fixation at a single academic institution. This study, approved by the Clinical Trial Review Committee, spanned from May 2018 to October 2022.

Inclusion criteria for LDD conditions encompassed lumbar disc herniation, degenerative lumbar scoliosis, stenosis, and synovial cysts. Our criteria for fusion surgery were >3 mm of sagittal translation or >10° segmental angulation angle on dynamic radiographic evaluation or posterior widening of the disc space of >5° on a flexion radiograph. Patients received a comprehensive preoperative diagnosis through medical history analysis, neurological and radiological examinations, myelography, CT-scanned myelography, and/or magnetic resonance imaging (MRI). Surgeons determined the stenosis location using preoperative images. Eligibility criteria for the study required patients to be over 18, experiencing low back pain, leg pain, and/or leg numbness, and non-responsive to at least three months of conservative treatment pre-surgery.

The study also targeted patients who underwent CT or MRI imaging evaluations pre- and post-surgery and who could be assessed using pain scores and the Japanese Orthopedic Association Back Pain Evaluation Questionnaire (JOABPEQ) one year postoperatively. Informed consent was obtained from each participant before surgery.

Out of 212 assessed patients, 109 (68 males and 41 females) met the inclusion criteria, representing a 50% inclusion rate. Table 1 provides detailed demographic data.

Baseline demographic data, including age, gender, height, weight, body mass index (BMI), smoking status, and steroid use, were collected. Surgical data, such as cage factors, duration, estimated total blood loss, and perioperative complications, were also documented. The average age was 69.8 ± 10.4 years.

### 2.3. Surgical Technique

We detail the fundamental technique for indirect decompression via LLIF and posterior fixation, as initially proposed by Ozgur et al. [24] and elaborated in our prior publications [25,26]. The LLIF procedure was executed using either the extreme lateral interbody fusion (XLIF) technique or the oblique lateral interbody fusion (OLIF) approach, with the specific method chosen at the surgeon’s discretion, as there were no set criteria for selection between the two. Upon analyzing the preoperative HU values, no statistically significant differences in bone density were found between patients in the XLIF and OLIF groups. Therefore, the choice of surgical technique did not influence the bone density outcomes in this study.

The LLIF was executed through a single-incision, mini-open method under direct visualization. This entailed positioning the patient laterally, making a skin incision near the targeted intervertebral disc, and accessing and removing the disc, followed by cage trials and insertions. For all LLIF segments, percutaneous pedicle screw fixation was applied, utilizing either intraoperative fluoroscopy or CT-based navigation.

### 2.4. Lumbar MRI

All patients underwent a standard lumbar MRI scan. The MRI scans, encompassing the lumbar region from L1–L2 to L5–S1, were conducted using T1- and T2-weighted sequences on either a 1.5 or 3.0 Tesla imaging system (Ingenia or Achieva models, Philips Medical Systems, Best, The Netherlands). The primary purpose of the MRI was to assess the midsagittal canal diameter (CD) and the axial central canal area (CCA) of the thecal sac both before surgery and within two weeks postoperatively.

### 2.5. CT Evaluation

We evaluated endplate injury and cage subsidence (CS) using X-ray and CT imaging. CS refers to the sinking of the interbody cage into the adjacent vertebral endplates postoperatively. We considered subsidence evident in X-rays and/or CT images taken during hospitalization and occurring within the first two weeks postoperatively as early cage subsidence (ECS). Conversely, if there was no evidence of endplate injury in X-rays or CT scans within the first two weeks post-surgery but subsidence was detected in later X-rays or CT scans, we classified it as delayed cage subsidence (DCS). In other words, DCS is characterized by the gradual sinking of the cage into the vertebral endplates beyond the initial postoperative period. Finally, we combined those with ECS and DCS, and we determined the number of patients with CS in one year postoperatively.

We used a lumbar radiographic examination for patients who could not undergo a CT scan one year after surgery. We defined fusion status from the CT scan as a bony bridge in the sagittal and coronal reconstruction planes, with its partial or complete connections to the lower and upper endplates. This means that our fusion criteria also included cases of partial fusion.

In contrast, we defined fusion in lateral dynamic radiography as the presence of regional motion of less than 3° and intervertebral translation of less than 3 mm, without having had a revision or evidence of instrumentation loosening one year after surgery. We classified the fusion status as pseudarthrosis if we found the cleft sign in any position.

### 2.6. Measurements of HU Value

Our study used previous research methods to measure HU values from preoperative CT scans, explicitly focusing on the L1 to L4 vertebrae. These scans were performed using one of four types of helical CT scanners by Siemens Healthineers (Erlangen, Germany): Somatom Definition Edge, Somatom X.cite, Somatom Force, or Naetom Alpha. The CT scan settings were standardized to a tube voltage of 120 kVp and a tube current of 280 mA, with a slice thickness of 3.0 mm and intervals set equally. Using the Picture Archiving and Communication System (PACS), we automatically computed the average HU values for the designated regions of interest (ROI). HU measurements were conducted in three specific areas: directly below the superior endplate, at the center of the vertebral body, and just above the inferior endplate [27].

The axial CT slices selected for HU value evaluation focused on areas rich in cancellous bone, intentionally avoiding cortical bone. We carefully chose the ROI for each anatomical structure, ensuring accurate conformity to the structure’s shape. Once a consistent and suitable ROI was established, the PACS was used to calculate the average HU values. We then determined the mean HU values from L1 to L4, defining this as the L1–L4 HU value.

This metric was employed in a cluster analysis to categorize subjects into two groups: those with low HU values and those with high HU values.

### 2.7. Pain Intensity

We utilized three distinct Numeric Rating Scales (NRS) to evaluate pain intensity in three specific areas: lower back pain (LBP), leg pain (LP), and leg numbness (LN) [28]. We conducted 11 assessments for each type of pain, using a scale ranging from 0, signifying an absence of pain, to 10, indicative of the most extreme pain imaginable. These assessments were performed both before the surgical procedure and one year after it. We employed the delta symbol (Δ) to quantify the variation in pain levels, comparing the scores post-surgery with those recorded pre-surgery.

### 2.8. JOABPEQ

We briefly detail the use of the JOABPEQ, a patient-centric instrument for assessing individuals’ QOL [29,30]. This tool evaluates five key areas (low back pain, lumbar function, walking ability, social life, and mental health) through answers to 25 questions. Score calculation can be performed via formulas provided or an Excel sheet from the Japanese Orthopedic Association’s website (v.070903, https://ssl.jssr.gr.jp/member/joa/, accessed on 13 October 2024). We carried out JOABPEQ assessments before and one year after the surgical procedure. The tool’s effectiveness was gauged using two main metrics: the effectiveness rate and the gained score.

The effectiveness rate was determined by either a minimum 20-point increase in a patient’s score from before to after surgery or by achieving a score of 90 or higher post-treatment. Instances where patients scored 100 points in both pre- and post-surgery were excluded from this calculation. The effectiveness rate was computed as the percentage of patients who satisfied these criteria among the total evaluated.

The gained score was ascertained by contrasting the pre-surgical and post-surgical scores.

### 2.9. Statistical Analysis

We conducted our statistical analysis using IBM SPSS Statistics software (version 23.0, IBM Corp., Armonk, NY, USA), reporting all values as mean ± standard deviation.

Initially, we performed a hierarchical cluster analysis to classify patients into two distinct groups based on the mean HU values from the L1 to L4 vertebrae: those with low HU values and those with high HU values. Following this, we applied PSM to pair individuals from these groups, ensuring demographic comparability. The propensity scores were calculated using a multivariable logistic regression model, which considered various patient demographics such as age, gender, height, body weight, and BMI. We employed the optimal matching method to minimize the disparity in propensity scores between matched pairs. The ideal caliper width for matching was determined as 0.2 times the standard deviation of the logit of the propensity scores, a method previously described by Austin [31].

For statistical analysis, we first evaluated the data of the two initial groups without applying propensity score matching. Subsequently, we compared the data from the new groups formed using the propensity score matching method. To assess the normality of our data, we used the Shapiro–Wilk test. We applied the chi-square test and Student’s *t*-test for normally distributed data, while for non-normally distributed data, we used the Mann–Whitney U test. A type 1 error threshold of 5% was set for all statistical tests, considering *p* values less than 0.05 to be indicative of statistical significance.

## 3. Results

This study evaluated HU measurements at various lumbar spine levels (L1, L2, L3, and L4) (Table 2).

**Figure 2:**
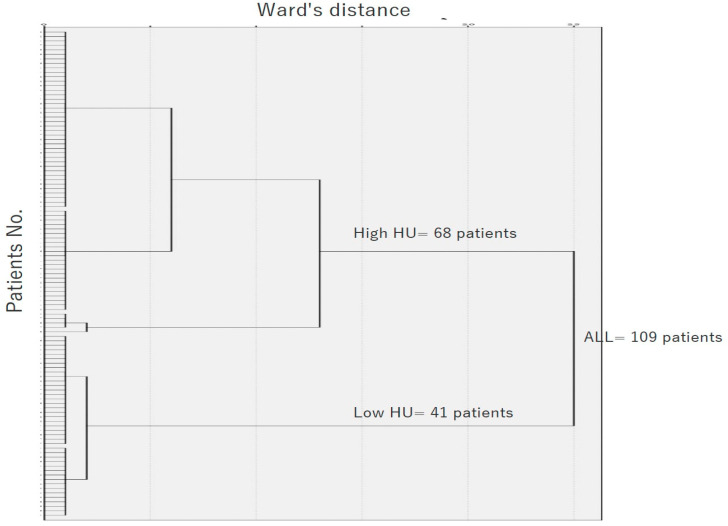
Hierarchical dendrogram of patients (*n* = 109) who underwent indirect decompression via LLIF. The dendrogram displays the number of patients in each cluster at varying distances between wards. Through cluster analysis, patients were categorized into a low HU group (*n* = 41) and a high HU group (*n* = 68). HU, Hounsfield unit.

At L1, the average HU was 132 ± 54, slightly higher at L2 with an average of 136 ± 61, and at L3, the average HU was 137 ± 61. The highest average HU was observed at L4, which was 149 ± 66. There was a trend of gradually increasing bone density from the superior to the inferior vertebrae, but no statistically significant differences were found within the vertebrae. Additionally, there were no statistically significant differences in the average HU values between the groups from L1 to L4 (*p* = 0.204). The overall average HU from L1 to L4 was 139 ± 51. Based on the cluster analysis results, patients were classified into low HU and high HU groups (Figure 2).

**Figure 3:**
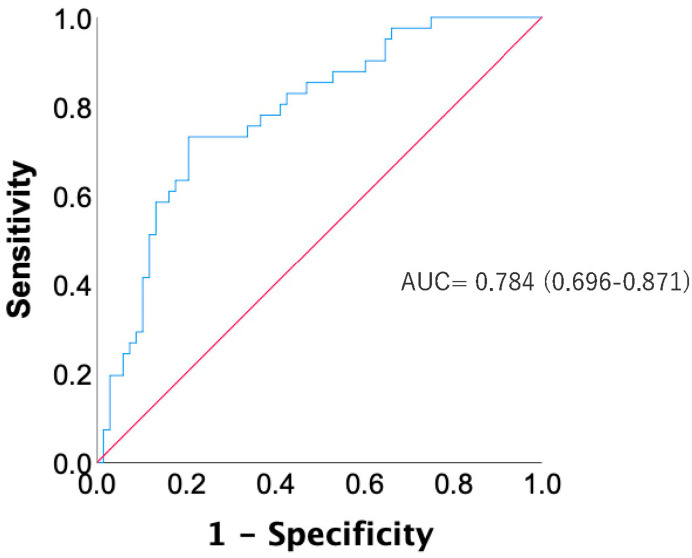
ROC curve showing the correlation between HU values (low HU and high HU) and propensity score. The blue line represents the ROC curve, illustrating the relationship between sensitivity (True Positive Rate) and 1—specificity (False Positive Rate) at various threshold settings. The red line is the diagonal line that represents a random classifier, where the model’s ability to discriminate is equivalent to chance (AUC = 0.5). It serves as a baseline for comparison with the blue ROC curve. HU, Hounsfield unit; ROC, receiver operator characteristic.

Initially, 41 patients were in the low HU group and 68 in the high HU group. Post-PSM, each group consisted of 26 patients. The area under the curve (AUC) from the Receiver Operating Characteristic (ROC) curve was 0.784 (0.696–0.871), validating the trend score’s efficacy in determining HU grouping (Figure 3).

Our study analyzed two distinct patient groups categorized by their HU values before and after PSM (Table 3).

Notable pre-PSM differences in age and sex distribution, with the high HU group being younger and predominantly male, were neutralized post-PSM, indicating effective group balancing. Post-PSM, no significant differences were found in BMI, height, body weight, or histories of tobacco and steroid use. Surgical indications and details, including treated lumbar segments and cages, were comparable between groups. Regarding complications, the overall complication rates were similar between the two groups post-PSM. Specifically, the incidence of transient motor weakness, thigh pain and/or numbness, and revision surgery did not differ significantly between the groups. However, it is noteworthy that CS rates were higher in the low HU group pre-PSM (34% vs. 15%, *p* = 0.030), but this difference was not significant post-PSM (35% vs. 19%, *p* = 0.349). These findings suggest that while initial differences in certain complications existed, the propensity score matching effectively balanced the groups, leading to comparable complication rates postoperatively. There was also no significant difference in bone fusion assessment with CT or X-ray between the two groups, with 20 patients (77%) in the low HU group and 19 patients (73%) in the high HU group, respectively, which was not statistically significant (*p* = 1.000).

Significant increases in both CD and CCA were observed post-surgery (Table 4).

In the low HU group, CD increased from 5.7 ± 2.4 mm preoperatively to 8.5 ± 2.3 mm postoperatively, and CCA from 61.9 ± 41.2 mm^2^ to 90.5 ± 35.3 mm^2^. The high HU group showed increases in CD from 6.1 ± 2.1 mm to 8.1 ± 1.8 mm and in CCA from 60.2 ± 34.5 mm^2^ to 80.9 ± 30.3 mm^2^. For all patients, CD increased from 5.9 ± 2.2 mm to 8.3 ± 2.0 mm and CCA from 61.0 ± 37.5 mm^2^ to 85.4 ± 32.8 mm^2^. All changes were statistically significant (*p* < 0.001), with no significant inter-group differences (CD: *p* = 0.102; CCA: *p* = 0.221).

**Figure 4:**
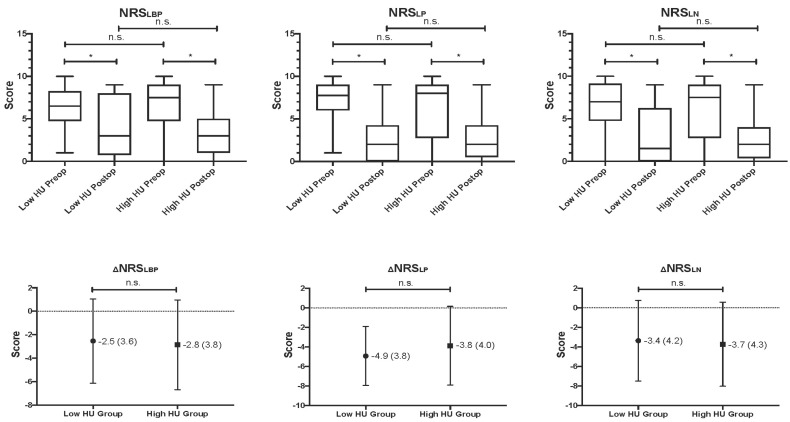
Comparison of pain intensity between the low and high HU groups. Pain intensity was measured using the NRS for three types of pain: low back pain (NRS_LBP_), leg pain (NRS_LP_), and leg numbness (NRS_LN_). The graph shows significant improvements in pain scores from preoperative to one-year postoperative assessments in both the low HU and high HU groups. No significant differences in pain reduction were observed between the two groups, indicating similar clinical improvements regardless of preoperative bone density. Error bars represent the standard deviation (*p* < 0.05 indicates significant differences; * Statistically significant.; n.s. indicates no significant difference). ΔNRS: Mean Pain Reduction by NRS.

The mean evaluation period for both clinical and radiographic assessments was 12.1 ± 1.4 months. Pain scores before and after surgery indicated significant improvements (Figure 4).

In the low HU group, the Numeric Rating Scale for Low Back Pain (NRS_LBP_) decreased from 6.2 to 3.7, and in the high HU group, from 6.5 to 3.6 (*p* = 0.001 for both). The overall group decreased from 6.4 to 3.7 (*p* < 0.001). Similarly, Numeric Rating Scale for Leg Pain (NRS_LP_) scores improved significantly in both subgroups. The low HU group’s scores decreased from 7.4 to 2.5, and the high HU group from 6.3 to 2.5 (*p* < 0.001). Both groups showed significant improvements for the Numeric Rating Scale for Leg Numbness (NRS_LN_), with the low HU group decreasing from 6.4 to 3.0 and the high HU group from 6.2 to 2.4. The overall group’s average decreased from 6.3 to 2.7. Comparisons at different time points showed no significant differences, indicating equal treatment effectiveness across HU classifications.

This study also focused on the effective rate of improvement and changes in JOABPEQ scores across five domains (Table 5).

In low back pain, the effective rate was 72% for the low HU group and 66.7% for the high HU group. In lumbar function, the effective rate was 24% and 19.2% for the low and high HU groups, respectively. The effective rate of walking ability was 80% for the low HU group and 73.1% for the high HU group. Both groups showed identical effective rates of 57.7% in social life function and 30.8% in mental health. No significant differences were observed between the low and high HU groups in these domains.

Both groups observed significant improvements in JOABPEQ scores post-surgery across all domains. Low back pain, walking ability, social life function, and mental health showed statistically significant improvements. However, lumbar function improved, though not significantly. In addition, score improvements were not significantly different between the low and high HU groups.

## 4. Discussion

In this study, we delved into the role of preoperative HU values in predicting short-term clinical outcomes post-LLIF surgery. Challenging previous assertions that low HU values are associated with increased risks of complications like cage subsidence [32], our findings, although based on short-term outcomes, indicate no significant differences in postoperative pain or QOL, regardless of the HU values. This revelation offers novel insights, especially in the realm of minimally invasive spinal surgery for patients with LDD.

Based on preoperative CT scans, we categorized patients into low HU and high HU groups. Furthermore, by employing the PSM method to standardize patient backgrounds, such as age, gender, height, weight, and BMI, we could compare and examine the impact of HU values on recovery after LLIF surgery. Contrary to earlier beliefs that low HU values predispose patients to risks such as cage subsidence [33], our study suggests a more complex relationship between bone quality and surgical success. Notably, both groups showed significant improvements in clinical outcomes, such as pain reduction and functional recovery, as assessed by the JOABPEQ score. These results suggest that, in the short term, BMD has little impact on postoperative pain or QOL. However, it should be noted that certain factors, including the surgical techniques employed and patient selection criteria, might have contributed to the outcomes observed and could introduce potential biases in our findings. Further refinement in surgical planning and patient stratification could optimize outcomes for specific patient groups with low bone density.

Our findings highlight the previously reported efficacy of LLIF in achieving indirect decompression without compromising the posterior supporting structures [34]. LLIF’s ability to reduce the mechanical stress on posterior elements is similar to the principles seen in non-fusion surgeries, such as those using isobar devices, which were shown by Guan et al. to reduce surgical time, blood loss, and the incidence of adjacent segment disease (ASD) [35]. While non-fusion techniques preserve lumbar mobility, LLIF also maintains stability with fewer invasive procedures. Therefore, in carefully selected cases, LLIF may provide benefits similar to non-fusion techniques but with the added assurance of achieving long-term segmental stability.

While prior studies have established a correlation between low HU values and an increased risk of postoperative complications [36], our analysis suggests these factors may not substantially affect short-term clinical outcomes when using LLIF for short fusion up to three levels. However, it is important to emphasize that while short-term outcomes such as pain relief and functional recovery were positive, longer-term studies are necessary to evaluate the true impact of HU values on complications such as cage subsidence and adjacent segment disease. In particular, identifying specific thresholds for HU values that might predict complications would enhance preoperative decision-making and risk stratification.

Furthermore, in line with previous reports, we emphasize the utility of HU measurements in spinal surgery as a practical alternative to conventional DXA assessments. By utilizing routine preoperative CT scans for HU evaluation, surgeons can gain a deeper understanding of bone quality, which aids in customizing surgical approaches to meet patients’ individual needs.

Nevertheless, the variability in HU cutoff values for diagnosing osteoporosis, as seen in the literature, suggests that there is no one-size-fits-all solution [37]. Developing a standardized approach to HU evaluation, particularly in combination with other diagnostic tools such as DXA, could improve the consistency and applicability of bone health assessments across institutions.

Despite its valuable insights, our study’s retrospective nature and single-institution setting might limit the generalizability of our results. Previous reports have associated lower HU values with an increased risk of postoperative complications [32,38,39]. However, our findings indicate that HU values in LLIF surgery do not significantly differ in postoperative pain scores or QOL. It is also essential to acknowledge the multifaceted nature of postoperative pain, which psychological factors or patient expectations could influence. A more holistic approach to postoperative pain management, integrating physical and mental health strategies, could enhance patient outcomes.

Additionally, our study, which focuses on one-year post-surgery outcomes, is limited by its short follow-up period. A long-term analysis is essential, particularly regarding adjacent segment disease after fixation surgery. Future research with longer follow-ups and a larger, more diverse cohort is necessary to validate and expand our findings. In particular, the need for a 2–5-year follow-up is critical, as ASD often manifests beyond the first year. Implementing routine postoperative imaging and patient-reported outcome measures (PROMs) at regular intervals would provide a clearer picture of long-term success and complications.

Furthermore, as the study included only 52 patients after propensity score matching, the conclusions drawn may only partially represent the broader population of patients undergoing LLIF surgery. Although the matching process reduces confounding factors, the relatively small sample size limits the statistical power and generalizability of the findings. Moreover, despite being labeled ‘short-term’, the short-term nature of the outcomes does not fully capture the long-term implications of HU values on spinal surgery outcomes. While this study observed improvements at one year, a long-term follow-up is necessary to comprehensively assess outcomes such as ASD, which often manifest beyond the first year post-surgery.

Finally, we also did not investigate the correlation between BMD as measured by DXA and HU values from CT scans. While the importance of lumbar spine BMD is acknowledged, not all patients underwent DXA. Díaz-Romero and colleagues highlighted that many surgeons neglect bone health assessment before spinal fusion surgery, potentially missing critical osteoporosis treatment before and after surgery [40]. Moreover, due to the retrospective nature of this study, we could not accurately capture the proportion of patients who received pre- or postoperative osteoporosis treatment, as external healthcare providers managed some. This limitation, combined with the challenges of consistently managing osteoporosis treatment in patients at university hospitals in Japan, where regular monthly follow-up is difficult, may have affected our ability to fully assess the role of bone health management in surgical outcomes. Despite these limitations, our research contributes to understanding the efficacy of LLIF for indirect decompression and could improve patient care in this specialized field.

## 5. Conclusions

This study reveals that while preoperative HU values indicate bone quality, they may not significantly affect short-term outcomes in pain and QOL following LLIF surgery up to three levels. The use of HU values from routine CT scans offers a practical approach for surgical planning in spinal surgery, particularly in cases of LDD. However, this study’s retrospective nature and limited scope suggest further research with longer follow-ups and broader patient demographics to fully understand the implications of HU values in spinal surgery outcomes.

## Figures and Tables

**Table 1:** Characteristics of the subjects in the present study.

Characteristic		Data
No. of patients		109
Age (years), mean (SD)		69.8 (10.4)
Sex (male/female), *n*		68/41
Height (cm), mean (SD)		160.1 (9.2)
Body weight (kg), mean (SD)		63.2 (12.1)
BMI (kg/m^2^), mean (SD)		24.5 (3.5)
Tobacco use, *n* (%)		26 (24)
Steroid use, *n* (%)		6 (6)
Indications, *n* (%)	LCS+ (LDS)	92 (84)
	LDH	6 (6)
	FS	5 (4)
	DLS	4 (4)
	Synovial cyst	2 (2)
Levels treated, *n* (%)	L1–L2	2 (1)
	L2–L3	13 (8)
	L3–L4	57 (37)
	L4–L5	84 (54)
	Overall	156 (100)
Number of fused segments, *n* (%)	1 level	68 (62)
	2 level	35 (32)
	3 level	6 (6)
	mean	1.4 (0.6)
OR time (min), mean (SD)		115.7 (35.5)
EBL (mL), mean (SD)		93.7 (122.2)
Length of stay (days), mean (range, SD)		15.5 (4.6)
ECS, *n* (%)		12 (11)
DCS *n* (%)		12 (11)
CS, *n* (%)		24 (22)
Transient motor weakness, *n* (%)		12 (11)
Thigh pain and/or numbness, *n* (%)		16 (15)
Revision surgery, *n* (%)		2 (2)

Data presented as mean (SD) or number of patients (%). BMI, body mass index; OR, operation; EBL, estimated blood loss; LCS, lumbar canal stenosis; LDS, lumbar degenerative spondylolisthesis; DLS, degenerative lumbar scoliosis; FS, foraminal stenosis; LDH, lumbar disc herniation; ECS, early cage subsidence; DCS, delayed cage subsidence; CS, cage subsidence.

**Table 2:** Results of HU measurements at L1, L2, L3, L4, and L1–L4. Data presented as mean (SD) HU: Hounsfield units.

Level	Position	HU (SD)
HU at L1	superior	119 (50)
	middle	126 (51)
	inferior	151 (82)
	mean	132 (54)
HU at L2	superior	124 (66)
	middle	123 (50)
	inferior	162 (87)
	mean	136 (61)
HU at L3	superior	130 (66)
	middle	126 (53)
	inferior	155 (87)
	mean	137 (61)
HU at L4	superior	138 (74)
	middle	134 (63)
	inferior	174 (91)
	mean	149 (66)
HU at L1–L4	mean	139 (51)

**Table 3:** Comparison of two groups.

		Before PSM			After PSM		
Variable		Low HU	High HU	*p* Value	Low HU	High HU	*p* Value
No. of patients, *n* (%)		41	68		26	26	
Hounsfield units		92 (18)	167 (43)	-	93 (18)	167 (39)	-
Propensity score					0.448 (0.212)	0.446 (0.217)	-
Age (years), mean (SD)		73.4 (6.0)	67.6 (11.9)	0.001 *	73.5 (6.1)	74.6 (5.6)	0.493
Sex (male/female), *n*		19/22	49/19	0.009 *	16/10	18/8	0.569
Height (cm), mean (SD)		158.3 (9.7)	161.2 (8.9)	0.115	159.1 (10.0)	160.0 (9.1)	0.750
Body weight (kg), mean (SD)		59.6 (10.3)	65.3 (12.6)	0.016 *	60.7 (11.6)	61.2 (10.0)	0.867
BMI (kg/m^2^), mean (SD)		23.8 (3.6)	25.0 (3.4)	0.081	24.0 (4.2)	23.9 (3.0)	0.919
Tobacco use, *n* (%)		9 (22)	17 (25)	0.818	8 (31)	3 (12)	0.106
Steroid use, *n* (%)		4 (10)	2 (3)	0.195	2 (8)	1 (4)	1.000
Indications, *n* (%)	LCS+ (LDS)	36 (88)	56 (82)	0.903	24 (92)	24 (92)	1.000
	LDH	1 (2)	5 (7)		0 (0)	0 (0)	
	FS	1 (2)	4 (6)		1 (4)	1 (4)	
	DLS	2 (5)	2 (3)		1 (4)	1 (4)	
	Synovial cyst	1 (2)	1 (1)		0 (0)	0 (0)	
Treated lumbar segments, *n* (%)	L1–L2	1 (2)	1 (1)	0.421	1 (3)	0 (0)	0.723
	L2–L3	4 (7)	9 (8)		3 (8)	2 (5)	
	L3–L4	17 (31)	40 (40)		12 (33)	16 (42)	
	L4–L5	33 (60)	51 (50)		20 (56)	20 (53)	
	Overall	55	101		36 (100)	38 (100)	
Operated spinal segments, *n* (%)	1 level	29 (71)	39 (57)	0.226	18 (69)	15 (58)	0.651
	2 level	10 (24)	25 (37)		6 (23)	10 (38)	
	3 level	2 (5)	4 (6)		2 (8)	1 (4)	
	mean	1.3 (0.6)	1.5 (0.6)		1.4 (0.6)	1.5 (0.6)	
	Overall	41	68		26 (100)	26 (100)	
Cage height (mm), *n* (%)	8.0–9.0	11 (27)	26 (38)	0.446	6 (17)	4 (11)	0.376
	9.0–10.0	23 (56)	36 (53)		17 (47)	17 (45)	
	10.0–11.0	17 (41)	32 (47)		11 (31)	13 (34)	
	11.0–	4 (10)	7 (10)		2 (6)	4 (11)	
	mean (SD)	9.4 (1.1)	9.2 (1.0)		9.3 (1.0)	9.5 (1.0)	
Cage width (mm), *n* (%)	18	55 (100)	101 (100)	-	36 (100)	38 (100)	-
Cage length (mm), *n* (%)	45	6 (11)	6 (6)	0.669	3 (8)	2 (5)	0.134
	50	11 (20)	32 (32)		7 (19)	16 (42)	
	55	34 (62)	59 (58)		22 (61)	19 (50)	
	60	4 (7)	4 (4)		4 (11)	1 (3)	
	mean (SD)	53.3 (3.9)	53.0 (3.3)		53.8 (3.8)	52.5 (3.2)	
OR time (min), mean (SD)		105.9 (27.6)	121.6 (38.5)	0.024 *	106.5 (30.9)	121.8 (31.5)	0.083
EBL (mL), mean (SD)		68.9 (68.1)	108.9 (144.2)	0.055	65.7 (76.1)	68.9 (56.9)	0.865
Length of stay (days), mean (SD)		15.5 (4.7)	15.5 (4.6)	0.968	15.5 (5.0)	16.0 (4.8)	0.758
ECS, *n* (%)		7 (17)	5 (7)	0.129	4 (15)	2 (8)	0.429
DCS, *n* (%)		7 (17)	5 (7)	0.129	5 (19)	3 (12)	0.476
CS, *n* (%)		14 (34)	10 (15)	0.030 *	9 (35)	5 (19)	0.349
Transient motor weakness, *n* (%)		3 (7)	9 (13)	0.529	3 (12)	4 (15)	1.000
Thigh pain and/or numbness, *n* (%)		3 (7)	13 (19)	0.104	1 (4)	7 (27)	0.050
Revision surgery, *n* (%)		0 (0)	2 (3)	1.000	0 (0)	1 (4)	1.000

Data presented as mean (SD) or number of patients (%). PSM, propensity score-matching; BMI, body mass index; OR, operation; EBL, estimated blood loss; LCS, lumbar canal stenosis; LDS, lumbar degenerative spondylolisthesis; DLS, degenerative lumbar scoliosis; FS, foraminal stenosis; LDH, lumbar disc herniation; ECS, early cage subsidence; DCS, delayed cage subsidence; CS, cage subsidence. * Statistically significant.

**Table 4:** Radiographic measures—canal dimension (canal diameter and central canal area of dural sac) changes evaluated on pre-and postoperative MRI.

		Preop	Postop	Change (Δ)	*p* Value ‡
CD (mm) mean (SD)	Low HU (*n* = 26)	5.7 (2.4)	8.5 (2.3)	2.8 (2.1)	<0.001 *
	High HU (*n* = 26)	6.1 (2.1)	8.1 (1.8)	2.0 (1.8)	<0.001 *
	ALL (*n* = 52)	5.9 (2.2)	8.3 (2.0)	2.4 (2.0)	<0.001 *
	*p* value †	0.500	0.410	0.102	
CCA (mm^2^) mean (SD)	Low HU (*n* = 26)	61.9 (41.2)	90.5 (35.3)	28.6 (26.8)	<0.001 *
	High HU (*n* = 26)	60.2 (34.5)	80.9 (30.3)	20.7 (25.2)	<0.001 *
	ALL (*n* = 52)	61.0 (37.5)	85.4 (32.8)	24.4 (26.0)	<0.001 *
	*p* value †	0.851	0.235	0.221	

Data presented as mean (SD). CD, canal diameter; CCA, central canal area. † Comparison with preop. ‡ Comparison between groups. * Statistically significant.

**Table 5:** Two-group comparison of pre- and postoperative JOABPEQ. (**A**) Two-group comparison of effective rate. Number of improved patients/number of patients (effective rate). (**B**) Two-group comparison of JOABPEQ score before and after surgery.

**(A)**					
		**Low HU** **(n = 26)**	**High HU** **(n = 26)**	**ALL** **(n = 52)**	***p* Value** **‡**
low back pain		18/25 (0.720)	16/24 (0.667)	34/49 (0.694)	0.762
lumbar function		6/25 (0.240)	5/26 (0.192)	11/51 (0.216)	0.743
walking ability		20/25 (0.800)	19/26 (0.731)	39/51 (0.765)	0.743
social life function		15/26 (0.577)	15/26 (0.577)	30/52 (0.577)	1.000
mental health		8/26 (0.308)	8/26 (0.308)	16/52 (0.308)	1.000
**(B)**					
		**Low HU** **(*n* = 26)**	**High HU** **(*n* = 26)**	**ALL** **(*n* = 52)**	***p*** **Value** **‡**
low back pain	Preop	43 (35.3 ± 27.6)	43 (38.6 ± 31.9)	43 (36.9 ± 29.5)	0.699
	Postop	71 (69.0 ± 27.2)	64 (63.3 ± 29.9)	71 (66.2 ± 28.4)	0.488
	*p* value †	<0.001 *	0.001	<0.001 *	
lumbar function	Preop	54 (52.4 ± 27.8)	54 (55.4 ± 24.5)	54 (53.9 ± 25.9)	0.686
	Postop	54 (57.5 ± 24.4)	62.5 (62.2 ± 25.9)	58 (59.9 ± 25.1)	0.521
	*p* value †	0.303	0.149	0.075	
walking ability	Preop	7 (15.2 ± 19.8)	21(24.1 ± 19.5)	14 (19.7 ± 20.0)	0.110
	Postop	50 (56.9 ± 26.6)	71 (60.2 ± 29.4)	64 (58.6 ± 27.8)	0.679
	*p* value †	<0.001 *	<0.001 *	<0.001 *	
social life function	Preop	31 (32.4 ± 21.7)	31 (32.4 ± 19.5)	31 (32.4 ± 20.4)	0.995
	Postop	51 (54.0 ± 22.4)	63.5 (58.6 ± 19.4)	58 (56.3 ± 20.9)	0.427
	*p* value †	<0.001 *	<0.001 *	<0.001 *	
mental health	Preop	42 (41.3 ± 17.4)	40.5 (41.3 ± 18.3)	42 (41.3 ± 17.7)	0.994
	Postop	55 (57.4 ± 18.7)	54 (52.7 ± 16.4)	54 (55.0 ± 17.6)	0.349
	*p* value †	<0.001 *	0.007 *	<0.001 *	

Score = median (mean ± S.D.). † Comparison with preop. ‡ Comparison between groups. * Statistically significant.

## Data Availability

The data are not publicly available due to privacy or ethical restrictions.

## References

[1] Wagner S.C., Formby P.M., Helgeson M.D., Kang D.G. (2016). Diagnosing the Undiagnosed: Osteoporosis in Patients Undergoing Lumbar Fusion. Spine.

[2] Xiao P.L., Cui A.Y., Hsu C.J., Peng R., Jiang N., Xu X.H., Ma Y.G., Liu D., Lu H.D. (2022). Global, regional prevalence, and risk factors of osteoporosis according to the World Health Organization diagnostic criteria: A systematic review and meta-analysis. Osteoporos. Int..

[3] Anderson P.A., Morgan S.L., Krueger D., Zapalowski C., Tanner B., Jeray K.J., Krohn K.D., Lane J.P., Yeap S.S., Shuhart C.R. (2019). Use of Bone Health Evaluation in Orthopedic Surgery: 2019 ISCD Official Position. J. Clin. Densitom..

[4] Maier G.S., Kolbow K., Lazovic D., Maus U. (2016). The Importance of Bone Mineral Density in Hip Arthroplasty: Results of a Survey Asking Orthopaedic Surgeons about Their Opinions and Attitudes Concerning Osteoporosis and Hip Arthroplasty. Adv. Orthop..

[5] Diebo B.G., Sheikh B., Freilich M., Shah N.V., Redfern J.A.I., Tarabichi S., Shepherd E.M., Lafage R., Passias P.G., Najjar S. (2020). Osteoporosis and Spine Surgery: A Critical Analysis Review. JBJS Rev..

[6] Chou S.H., LeBoff M.S. (2017). Vertebral Imaging in the Diagnosis of Osteoporosis: A Clinician’s Perspective. Curr. Osteoporos. Rep..

[7] Pacheco E.M., Harrison E.J., Ward K.A., Lunt M., Adams J.E. (2002). Detection of osteoporosis by dual energy X-ray absorptiometry (DXA) of the calcaneus: Is the WHO criterion applicable?. Calcif. Tissue Int..

[8] Pinto E.M., Neves J.R., Teixeira A., Frada R., Atilano P., Oliveira F., Veigas T., Miranda A. (2022). Efficacy of Hounsfield Units Measured by Lumbar Computer Tomography on Bone Density Assessment: A Systematic Review. Spine.

[9] Lenchik L., Weaver A.A., Ward R.J., Boone J.M., Boutin R.D. (2018). Opportunistic Screening for Osteoporosis Using Computed Tomography: State of the Art and Argument for Paradigm Shift. Curr. Rheumatol. Rep..

[10] Schreiber J.J., Anderson P.A., Hsu W.K. (2014). Use of computed tomography for assessing bone mineral density. Neurosurg. Focus.

[11] Zaidi Q., Danisa O.A., Cheng W. (2019). Measurement Techniques and Utility of Hounsfield Unit Values for Assessment of Bone Quality Prior to Spinal Instrumentation: A Review of Current Literature. Spine.

[12] Oliveira L., Marchi L., Coutinho E., Pimenta L. (2010). A radiographic assessment of the ability of the extreme lateral interbody fusion procedure to indirectly decompress the neural elements. Spine.

[13] Rabau O., Navarro-Ramirez R., Aziz M., Teles A., Mengxiao Ge S., Quillo-Olvera J., Ouellet J. (2020). Lateral Lumbar Interbody Fusion (LLIF): An Update. Glob. Spine J..

[14] Ricciardi L., Piazza A., Capobianco M., Della Pepa G.M., Miscusi M., Raco A., Scerrati A., Somma T., Lofrese G., Sturiale C.L. (2023). Lumbar interbody fusion using oblique (OLIF) and lateral (LLIF) approaches for degenerative spine disorders: A meta-analysis of the comparative studies. Eur. J. Orthop. Surg. Traumatol..

[15] Formica M., Quarto E., Zanirato A., Mosconi L., Vallerga D., Zotta I., Baracchini M.L., Formica C., Felli L. (2020). Lateral Lumbar Interbody Fusion: What Is the Evidence of Indirect Neural Decompression? A Systematic Review of the Literature. HSS J..

[16] Kirnaz S., Navarro-Ramirez R., Gu J., Wipplinger C., Hussain I., Adjei J., Kim E., Schmidt F.A., Wong T., Hernandez R.N. (2020). Indirect Decompression Failure After Lateral Lumbar Interbody Fusion-Reported Failures and Predictive Factors: Systematic Review. Glob. Spine J..

[17] Ponnusamy K.E., Iyer S., Gupta G., Khanna A.J. (2011). Instrumentation of the osteoporotic spine: Biomechanical and clinical considerations. Spine J..

[18] Lee S.J., Binkley N., Lubner M.G., Bruce R.J., Ziemlewicz T.J., Pickhardt P.J. (2016). Opportunistic screening for osteoporosis using the sagittal reconstruction from routine abdominal CT for combined assessment of vertebral fractures and density. Osteoporos. Int..

[19] Lee S.J., Graffy P.M., Zea R.D., Ziemlewicz T.J., Pickhardt P.J. (2018). Future Osteoporotic Fracture Risk Related to Lumbar Vertebral Trabecular Attenuation Measured at Routine Body CT. J. Bone Miner. Res..

[20] Hiyama A., Sakai D., Katoh H., Sato M., Watanabe M. (2022). Relationship between Hounsfield Units of Upper Instrumented Vertebrae, Proximal Junctional Failure, and Global Alignment and Proportion Score in Female Patients with Adult Spinal Deformity. World Neurosurg..

[21] Hiyama A., Sakai D., Katoh H., Sato M., Watanabe M. (2023). Comparative Analysis of Hounsfield Units and Vertebral Bone Quality Scores for Predicting Proximal Junctional Failure in Female Adult Spinal Deformity Patients Undergoing Planned 2-Stage Corrective Surgery with Lateral Lumbar Interbody Fusion. World Neurosurg..

[22] Mikula A.L., Lakomkin N., Pennington Z., Pinter Z.W., Nassr A., Freedman B., Sebastian A.S., Abode-Iyamah K., Bydon M., Ames C.P. (2022). Association between lower Hounsfield units and proximal junctional kyphosis and failure at the upper thoracic spine. J. Neurosurg. Spine.

[23] Yao Y.C., Chao H., Kao K.Y., Lin H.H., Wang S.T., Chang M.C., Liu C.L., Chou P.H. (2023). CT Hounsfield unit is a reliable parameter for screws loosening or cages subsidence in minimally invasive transforaminal lumbar interbody fusion. Sci. Rep..

[24] Ozgur B.M., Aryan H.E., Pimenta L., Taylor W.R. (2006). Extreme Lateral Interbody Fusion (XLIF): A novel surgical technique for anterior lumbar interbody fusion. Spine J..

[25] Hiyama A., Katoh H., Nomura S., Sakai D., Watanabe M. (2021). Intraoperative computed tomography-guided navigation versus fluoroscopy for single-position surgery after lateral lumbar interbody fusion. J. Clin. Neurosci..

[26] Hiyama A., Katoh H., Sakai D., Watanabe M. (2021). A New Technique that Combines Navigation-Assisted Lateral Interbody Fusion and Percutaneous Placement of Pedicle Screws in the Lateral Decubitus Position with the Surgeon Using Wearable Smart Glasses: A Small Case Series and Technical Note. World Neurosurg..

[27] Schreiber J.J., Anderson P.A., Rosas H.G., Buchholz A.L., Au A.G. (2011). Hounsfield units for assessing bone mineral density and strength: A tool for osteoporosis management. J. Bone Jt. Surg. Am..

[28] Hiyama A., Katoh H., Nomura S., Sakai D., Watanabe M. (2022). The Effect of Preoperative Neuropathic Pain and Nociceptive Pain on Postoperative Pain Intensity in Patients with the Lumbar Degenerative Disease Following Lateral Lumbar Interbody Fusion. World Neurosurg..

[29] Fukui M., Chiba K., Kawakami M., Kikuchi S., Konno S., Miyamoto M., Seichi A., Shimamura T., Shirado O., Taguchi T. (2008). Japanese Orthopaedic Association Back Pain Evaluation Questionnaire. Part 3. Validity study and establishment of the measurement scale: Subcommittee on Low Back Pain and Cervical Myelopathy Evaluation of the Clinical Outcome Committee of the Japanese Orthopaedic Association, Japan. J. Orthop. Sci..

[30] Fukui M., Chiba K., Kawakami M., Kikuchi S., Konno S., Miyamoto M., Seichi A., Shimamura T., Shirado O., Taguchi T. (2007). Japanese Orthopaedic Association Back Pain Evaluation Questionnaire. Part 2. Verification of its reliability: The Subcommittee on Low Back Pain and Cervical Myelopathy Evaluation of the Clinical Outcome Committee of the Japanese Orthopaedic Association. J. Orthop. Sci..

[31] Austin P.C. (2011). Optimal caliper widths for propensity-score matching when estimating differences in means and differences in proportions in observational studies. Pharm. Stat..

[32] Guha D., Mushlin H.M., Muthiah N., Vodovotz L.L., Agarwal N., Alan N., Hamilton D.K., Okonkwo D.O., Kanter A.S. (2022). Computed Tomography Hounsfield Units as a Predictor of Reoperation and Graft Subsidence After Standalone and Multilevel Lateral Lumbar Interbody Fusion. World Neurosurg..

[33] Amorim-Barbosa T., Pereira C., Catelas D., Rodrigues C., Costa P., Rodrigues-Pinto R., Neves P. (2022). Risk factors for cage subsidence and clinical outcomes after transforaminal and posterior lumbar interbody fusion. Eur. J. Orthop. Surg. Traumatol..

[34] Hiyama A., Katoh H., Sakai D., Tanaka M., Sato M., Watanabe M. (2020). Short-term comparison of preoperative and postoperative pain after indirect decompression surgery and direct decompression surgery in patients with degenerative spondylolisthesis. Sci. Rep..

[35] Guan J., Liu T., Li W., Zhao H., Yang K., Li C., Feng N., Jiang G., Yang Y., Yu X. (2022). Effects of posterior lumbar nonfusion surgery with isobar devices versus posterior lumbar interbody fusion surgery on clinical and radiological features in patients with lumbar degenerative diseases: A meta-analysis. J. Orthop. Surg. Res..

[36] Taniwaki H., Hoshino M., Kinoshita Y., Matsumura A., Namikawa T., Kato M., Takahashi S., Nakamura H. (2023). Lower preoperative Hounsfield unit values as a risk factor for poor 5-year clinical outcomes after lumbar spine surgery. Eur. Spine J..

[37] Ullrich B.W., Schwarz F., McLean A.L., Mendel T., Kaden I., Hein E., Lattauschke A., Beyer J., Hofmann G.O., Klauke F. (2022). Inter-Rater Reliability of Hounsfield Units as a Measure of Bone Density: Applications in the Treatment of Thoracolumbar Fractures. World Neurosurg..

[38] Pinter Z.W., Bernatz J., Mikula A.L., Lakomkin N., Pennington Z.A., Michalopoulos G.D., Nassr A., Freedman B.A., Bydon M., Fogelson J. (2024). Paraspinal Sarcopenia and Lower Hounsfield Units are Independent Predictors of Increased Risk for Proximal Junctional Complications Following Thoracolumbar Fusions Terminating in the Upper Thoracic Spine. Glob. Spine J..

[39] Ran L., Xie T., Zhao L., Huang S., Zeng J. (2022). Low Hounsfield units on computed tomography are associated with cage subsidence following oblique lumbar interbody fusion (OLIF). Spine J..

[40] Díaz-Romero Paz R., Sosa Henríquez M., Armas Melián K., Coloma Valverde G. (2019). Trends and attitudes of spine surgeons regarding osteoporosis [Tendencias de actuación de los cirujanos de columna respecto a la osteoporosis]. Neurocirugia.

